# Light Quality Modulates Photosynthesis and Antioxidant Properties of *B. vulgaris* L. Plants from Seeds Irradiated with High-Energy Heavy Ions: Implications for Cultivation in Space

**DOI:** 10.3390/plants11141816

**Published:** 2022-07-10

**Authors:** Ermenegilda Vitale, Luigi Gennaro Izzo, Chiara Amitrano, Violeta Velikova, Tsonko Tsonev, Palma Simoniello, Veronica De Micco, Carmen Arena

**Affiliations:** 1 Department of Biology, University of Naples Federico II, Via Cinthia, 80126 Naples, Italy; ermenegilda.vitale@unina.it; 2 Department of Agricultural Sciences, University of Naples Federico II, Via Università 100, 80055 Portici, Italy; luigigennaro.izzo@unina.it (L.G.I.); chiara.amitrano@unina.it (C.A.); demicco@unina.it (V.D.M.); 3 Institute of Plant Physiology and Genetics, Bulgarian Academy of Sciences, Acad. G. Bonchev Str., bl. 21, 1113 Sofia, Bulgaria; violet@bio21.bas.bg (V.V.); ttsonev@bio21.bas.bg (T.T.); 4 Department of Science and Technology, Parthenope University of Naples, Via Acton 38, 80133 Naples, Italy; palma.simoniello@uniparthenope.it; 5 BAT Center—Center for Studies on Bioinspired Agro-Environmental Technology, 80055 Portici, Italy

**Keywords:** antioxidants, *Beta vulgaris* L., ionizing radiation, light quality, photosynthesis, Space Closed Ecosystem

## Abstract

*Beta vulgaris* L. is a crop selected for cultivation in Space for its nutritional properties. However, exposure to ionizing radiation (IR) can alter plant photosynthetic performance and phytochemical production in the extraterrestrial environment. This study investigated if plant growth under different light quality regimes (FL—white fluorescent; RGB—red–green–blue; RB—red–blue) modifies the photosynthetic behavior and bioactive compound synthesis of plants sprouted by dry seeds irradiated with carbon or titanium high-energy ions. The study evidenced that: (i) the plant response depends on the type of heavyion; (ii) control and C-ion-irradiated plants were similar for photosynthetic pigment content and PSII photochemical efficiency, regardless of the LQ regime; (iii) under FL, net photosynthesis (A_N_) and water use efficiency (iWUE) declined in C- and Ti-ion plants compared to control, while the growth of irradiated plants under RGB and RB regimes offset these differences; (iv) the interaction Ti-ion× RB improved iWUE, and stimulated the production of pigments, carbohydrates, and antioxidants. The overall results highlighted that the cultivation of irradiated plants under specific LQ regimes effectively regulates photosynthesis and bioactive compound amounts in leaf edible tissues. In particular, the interaction Ti-ion × RB improved iWUE and increased pigments, carbohydrates, and antioxidant content.

## 1. Introduction

The realization of Bioregenerative Life Support Systems (BLSSs) is crucial in considering future long-term human-crewed missions in Space. Transit vehicles, space stations, and platforms on Moon and Mars will include self-sustaining artificial eco-systems based on the balance between heterotrophs (humans and microorganisms) and autotrophs (plants or algae). In particular, higher plants significantly contribute to re-storing the resources in closed environments, regenerating and purifying air through CO_2_ absorption and O_2_ evolution and transpiration, as well as producing fresh food supplies for the crew [1,2,3,4].

Space is a harsh environment compared to Earth. Many factors may constrain the plant’s survival in the extraterrestrial environment, including altered gravity, the interaction between microgravity and fluid dynamics, ionizing radiation (IR), and modified pressure and temperature conditions. Among space factors, ionizing radiation represents the main hazard for the survival of life forms, including plants, in exploratory-class missions [5].

Understanding the effects of IR on photosynthetic apparatus and, in general, on plant metabolism is a prerequisite for cultivating plants in Space. IR may affect the photosynthetic process at different levels: from molecular, impacting light-harvesting complexes, reaction centers, and electron transport carriers, to physiological level by affecting primary and secondary photosynthetic metabolism, also through anatomical changes of leaf structure [6,7,8,9]. In addition, the radio-induced stress in plants triggers the production of a large variety of antioxidant compounds which are engaged in the detoxification of reactive oxygen species (ROS) and, at the same time, enriches the nutritional properties of plant tissues [4,10,11,12].

Generally, plant responses to IR depend on several variables, including species, phenological stage at the time of irradiation, dose, and radiation quality [5,6,13]. The space radiation environment consists of a wide variety of ion species with a continuous range of energies. The principal galactic cosmic rays (GCR) include high-energy protons, alpha particles, and heavy ions (HZE—high-energy nuclei component). Therefore, testing plant response to specific ions at proper acute doses is a vital prerequisite to assessing plant radiosensitivity and evaluating the suitability of different crops for cultivation in Space.

Carbon (C) and titanium (Ti) are among the heavy ions considered representative of HZE and are often used to simulate the GRC spectrum in ground-based experiments [14,15]. However, very little is known about titanium conversely to carbon ions. Early studies on animal models evidence that Ti-ions induce oxidative stress and genomic alterations associated with several health risks [16,17,18]. In plants, Ti-ions have been reported to improve starch mobilization towards actively growing tissues of eye bean seedlings and stimulate the production of antioxidants [19].

Therefore, the defining agricultural practices, as well as micro-environmental parameters, are essential for the selection of suitable crops for space farming. For example, plants have different requirements for light intensity, quality, and duration [3,20]. In particular, the light spectral composition affects not only germination [21], plant architecture, and leaf anatomy [22,23,24] but also physiological processes [25], such as stomatal opening regulation [26], photosynthesis [27,28,29], pigment synthesis [30,31], and ultimately biomass production [20,32,33,34]. Furthermore, specific light quality treatments during growth may also stimulate the resistance to diseases and abiotic stress (high temperature, nutritional deficiency, and heavy metals) [27,35,36,37], improving the synthesis of antioxidants [38] which, in turn, can enhance the nutritional quality of crops [39]. 

Based on this evidence, the modulation of the light spectrum is a promising tool for improving plant productivity in space farming [40,41,42,43], also representing a means to face the constraints of the space environment.

From this perspective, studying the interaction between space IR and light quality (LQ) is gaining interest in space research. Recent studies on crops evidenced that the interplay LQ/IR may elicit essential plant traits, such as dwarf growth, increased photosynthesis and nutritional value [44,45]. The present study aimed to deepen the knowledge of the interaction between high-LET (Linear Energy Transfer) ionizing radiation and LQ on chard (*B. vulgaris* L. var. cicla) plants focusing specifically on the photosynthetic process to assess the suitability of this species to be cultivated in Bioregenerative Life Support Systems (BLSSs). Chard was chosen for this study because it is considered a functional food [46], and for the high nutritional value of its leaves, rich in healthy secondary metabolites. Furthermore, its compact size and the great amount of edible biomass make it suitable for Space cultivation [3]. 

The specific aims of this study were to investigate: (i) how the exposure of chard seeds C and Ti heavy ions, representative particles of the galactic cosmic rays, may affect the photosynthetic metabolism of chard; (ii) how plant development under specific LQ regimes may modify the photosynthetic response to C and Ti irradiation; (iii) if and at what level the interaction between IR and specific LQ treatments may promote the production of functional metabolites which are beneficial as a supplement for the astronauts’ diet. 

## 2. Results

### 2.1. Germination and Plant Biomass

**Figure 1:**
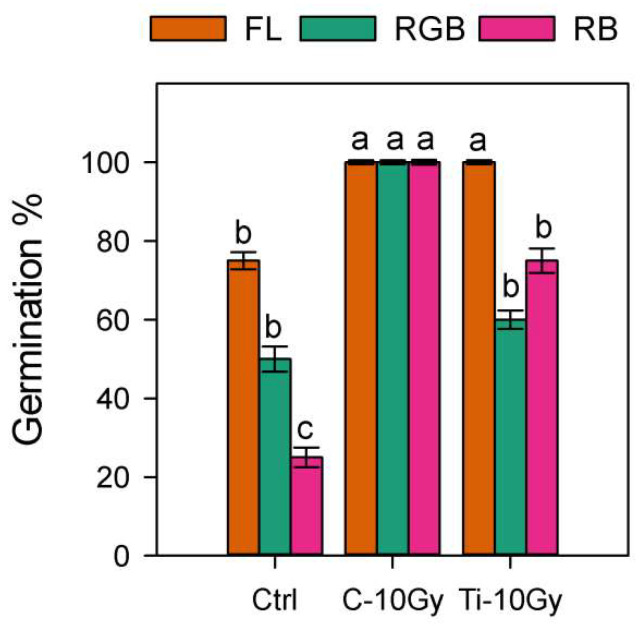
Percentage germination (GP) of Control (Ctrl) and irradiated Carbon (C-10 Gy) and Titanium (Ti-10 Gy) seeds of *B. vulgaris* under white fluorescent (FL), red–green–blue (RGB) and red–blue (RB) light quality regimes (*n* = 50). Different letters indicate statistically significant differences among treatments according to one-way ANOVA (*p* < 0.05).

Irradiation with C- and Ti-ions caused a significant increase in GP (100%) under FL light compared to not-irradiated control. RB light also determined a significant increase in GP after irradiation with C- (100%) and Ti-ions (75%). The same tendency to increase GP after irradiation was found under RGB light, but the values were significantly higher only in the case of C-ions (100%) compared to Ti and control treatments (Figure 1).

IR and LQ as the main factors did not significantly affect total biomass (TB) and shoot biomass (SB) (Table 1). Contrarily, the interaction IR × LQ was significant. More specifically, the RB regime induced an increase in TB and SB (*p* < 0.05) in plants from irradiated seeds (C 10-RB, Ti 10-RB) compared to the control (Ctrl-RB).

### 2.2. Gas Exchanges and Chlorophyll Fluorescence Emission Measurements

**Figure 2:**
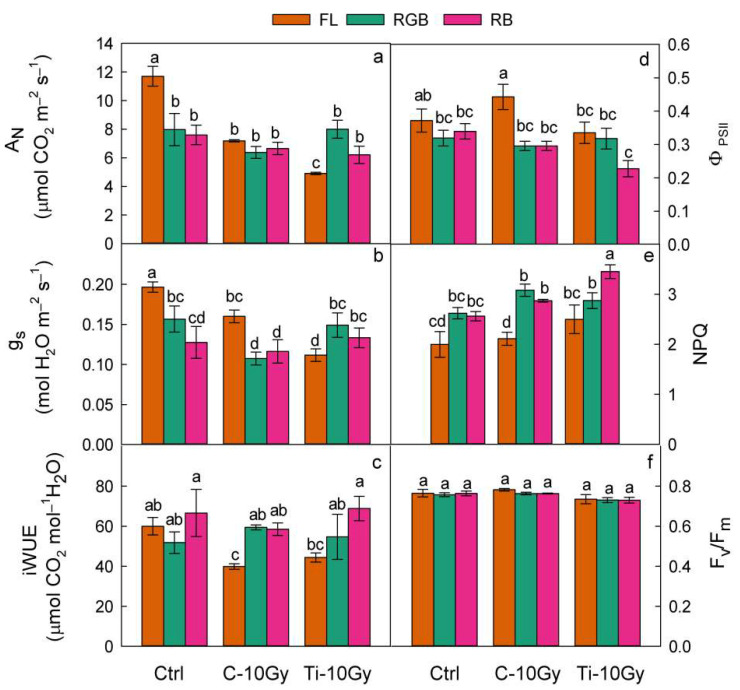
(**a**) Net CO_2_ assimilation, A_N_; (**b**) stomatal conductance, g_S_; (**c**) intrinsic water use efficiency, iWUE; (**d**) quantum yield of PSII electron transport, ϕ_PSII_; (**e**) non-photochemical quenching, NPQ; (**f**) maximum PSII photochemical efficiency, F_v_/F_m_ of *B. vulgaris* plants sprouted from Control (Ctrl) and irradiated Carbon (C-10 Gy), and Titanium (Ti-10 Gy) seeds and grown under white fluorescent (FL), red–green–blue (RGB) and red-blue (RB) light quality regimes (*n* = 5). Different letters indicate statistically significant differences among light treatments according to one-way ANOVA (*p* < 0.05).

The IR and LQ regimes strongly affected the photosynthetic performance of *B. vulgaris* plants as single factors and in combination. A_N_ and g_S_ of C-10 and Ti-10 plants declined (*p* < 0.01) and NPQ were raised (*p* < 0.05), while iWUE, ϕ_PSII_, and F_v_/F_m_ did not change significantly compared to control (Table 2). LQ, as a single factor, reduced A_N_ (*p* < 0.05), g_S_, and ϕ_PSII_, (*p* < 0.01) in RGB and RB compared to FL plants and an increased iWUE(*p* < 0.05) and NPQ (*p* < 0.001) (Table 2). No variation was observed in F_v_/F_m_ regardless of the IR treatments and LQ regimes (Table 2, Figure 2f).

The interaction IR × LQ (under FL regime) showed a significant decline of A_N_ (*p* < 0.001), g_S_ (*p* < 0.001), and iWUE (*p* < 0.05) in both C-10- and Ti-10-irradiated plants compared to control (Figure 2a–c). 

Growth under RGB and RB regimes did not induce any differences in A_N_ and iWUE among irradiated plants and respective controls (Figure 2a–c), while g_S_ showed the lowest (*p* < 0.01) value in C10-RGB plants (Figure 2b). 

Within the C-10 plant group, RGB and RB regimes reduced (*p* < 0.001) A_N_ and g_S_ compared to FL but did not influence iWUE (Figure 2a–c). In C-10 plants, A_N_ was unaffected by LQ, while RGB and RB regimes induced a decline (*p* < 0.001) in g_S_ and an increase (*p* < 0.05) of iWUE compared to FL (Figure 2a–c). Within the Ti-10 plant group, the RGB and RB light regimes enhanced A_N_ (*p* < 0.05), g_S_ (*p* < 0.05), and iWUE (*p* < 0.01) compared to FL (Figure 2a–c). 

In all LQ regimes, ϕ_PSII_ and F_v_/F_m_ ratio were not significantly affected by ionizing radiation. Contrary, among RB plants, Ti-ions significantly increased NPQ (Figure 2d–f). Within control plants, LQ regimes did not affect chard photochemistry. On the contrary, within the C-10 and Ti-10 plant groups, the RGB and RB regimes reduced (*p* < 0.01) ϕ_PSII_ and increased (*p* < 0.01) NPQ compared to FL. The strongest reduction of ϕ_PSII_ and the highest rise of NPQ were measured in Ti-10 RB plants (Figure 2d–f).

### 2.3. Plants Nutritional Traits and Bioactive Compounds

IR treatments and LQ regimes, as a single factor or interaction, determined a substantial variation in the concentration of photosynthetic pigment, total carbohydrate, proteins, and antioxidants (Table 3).

IR induced an increase (*p* < 0.001) in CHL and CAR concentration and a reduction (*p* < 0.01) in POL and ANTH in Ti-10 compared to Ctrl and C-10 plants. On the contrary, compared to control, C-10 plants showed comparable concentration of CHL, CAR, POL, and ANTH, lower (*p* < 0.05) CARB and PROT content, and higher (*p* < 0.001) level of TAC.

LQ as a single factor determined a reduction (*p* < 0.001) in CHL, CAR, and CARB under RGB and RB regimes (*p* < 0.001) compared to FL (Table 3), while for PROT and POL, the RGB plant group showed the lowest value. No significant difference was detected between FL and RB regimes. However, for ANTH and TAC, the different LQ regimes exerted diverse responses: RGB reduced (*p* < 0.01) ANTH content compared to FL and RB but increased TAC (*p* < 0.01).

**Figure 3:**
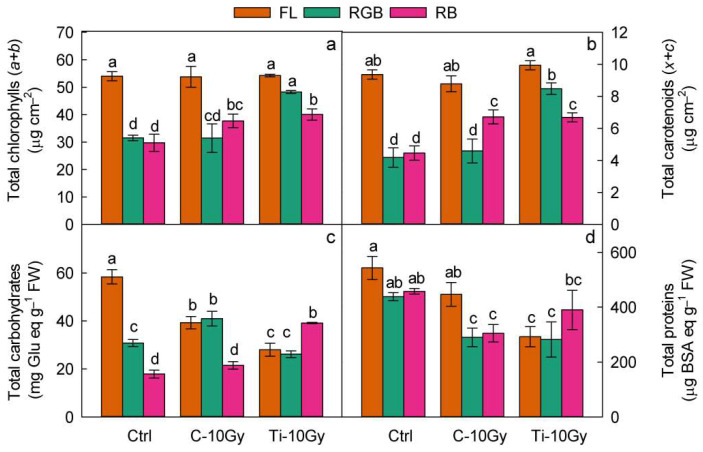
(**a**) Total chlorophylls; (**b**) total carotenoids; (**c**) total carbohydrates; (**d**) total proteins of *B. vulgaris* plants sprouted from Control (Ctrl) and irradiated Carbon (C-10 Gy), and Titanium (Ti-10 Gy) seeds and grown under white fluorescent (FL), red–green–blue (RGB) and red–blue (RB) light quality regimes (*n* = 5). Different letters indicate statistically significant differences among treatments according to one-way ANOVA (*p* < 0.05).

The analysis of interactions IR × LQ highlighted that both control and irradiated plants are characterized by lower (*p* < 0.001) CHL and CAR content under RGB and RB than under FL regime (Figure 3a,b).

Total carbohydrate content was affected by IR and LQ; indeed, it decreased (*p* < 0.05) in irradiated plants under the FL regime compared to control, while under RB, in Ti-10 plants, it was higher than in control (Figure 3c). C-FL plants showed the highest (*p* < 0.001) concentration of carbohydrates. In irradiated C-10 plants, the highest increase (*p* < 0.001) of carbohydrates was obtained under RGB regime, whereas within the Ti-10 plant group, the highest (*p* < 0.001) carbohydrate concentration was determined under the RB regime (Figure 3c).

The interaction IR × LQ consistently affected the protein content. Within the control and Ti-10 plant group, the protein content was not affected by LQ regimes. On the contrary, in the C-10 Gy plant group, the protein amount declined (*p* < 0.05) under RGB and RB compared to FL (Figure 3d). 

The comparison among control and irradiated plants at the same LQ regimes showed that the RGB reduced (*p* < 0.05) the total protein content in both irradiated plants compared to control. Otherwise, under FL and RB, the total protein concentration decreased only in Ti-10-FL (*p* < 0.05) and C-10-RB (*p* < 0.05) plants, respectively (Figure 3d).

**Figure 4:**
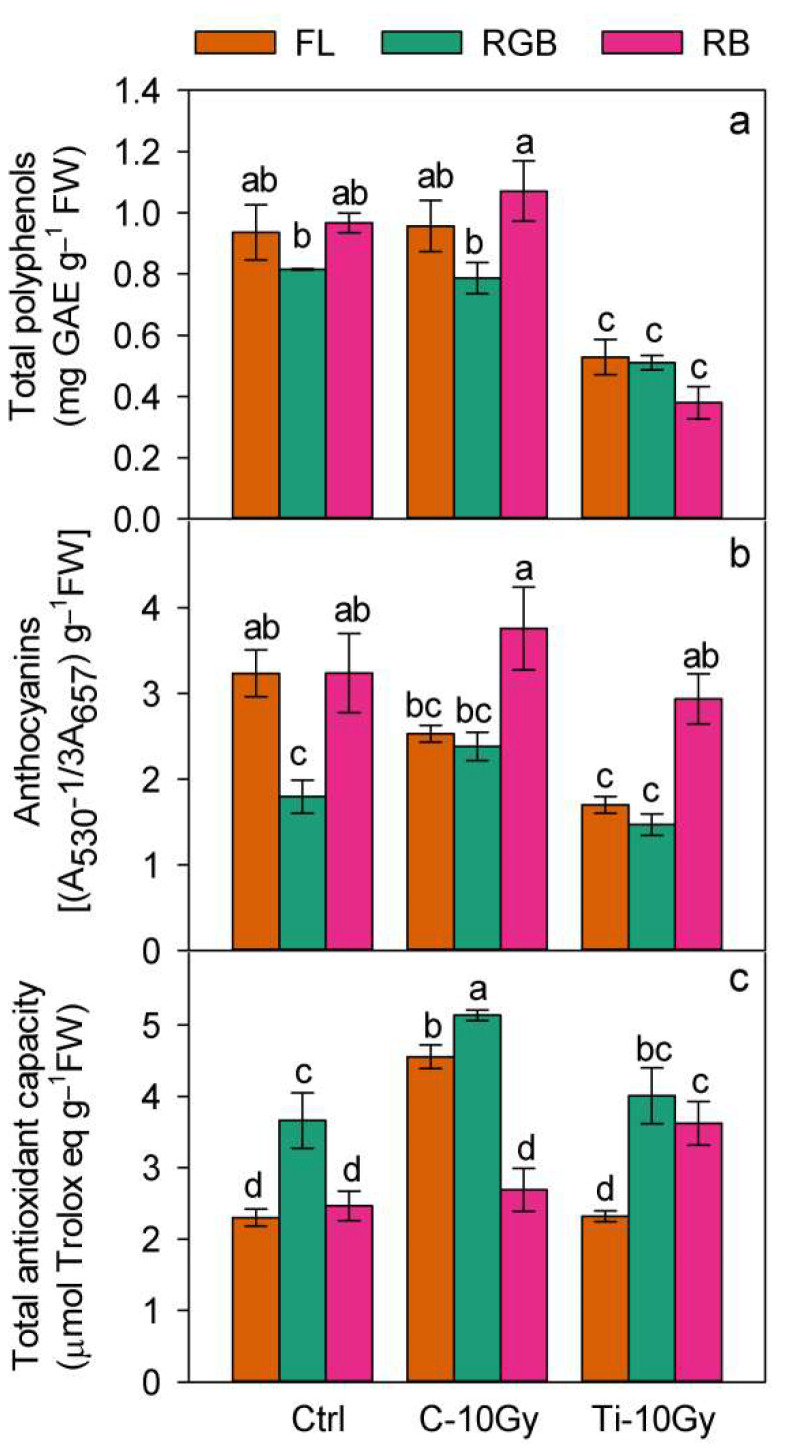
(**a**) Total polyphenol; (**b**) anthocyanin content; (**c**) total antioxidant capacity of *B. vulgaris* plants sprouted from Control (C) and irradiated Carbon (C-10 Gy), and Titanium (Ti-10 Gy) seeds and grown under white fluorescent (FL), red–green–blue (RGB) and red–blue (RB) light quality regimes (*n* = 5). Different letters indicate statistically significant differences among treatments according to one-way ANOVA (*p* < 0.05).

The interaction IR × LQ indicated that polyphenols significantly declined (*p* < 0.001) in T-10 plants regardless of LQ quality (Figure 4a). Under the FL regime, anthocyanins decreased (*p* < 0.01) in T-10-FL plants compared to Ctrl-FL plants. Conversely, under RGB and RB regimes, no significant difference in anthocyanin level was found between control and irradiated plants (Figure 4b). In the Ctrl plant group, anthocyanin concentration was lower (*p* < 0.05) under RGB than under FL and RB, whereas in C-10 and Ti-10 irradiated groups, anthocyanin levels increased under RB compared to FL and RGB regimes (Figure 4b).

Finally, the total antioxidant capacity within the control group increased (*p* < 0.05) under the RGB compared to FL and RB plants; in the C-10 plant group, the highest (*p* < 0.01) value was measured under the RGB regime, while in the Ti-10 plant group, the highest values were found under both RGB and FL (Figure 4c). The comparison among control and irradiated plants under the same LQ regime evidenced that under FL and RGB, C-10 plants showed the highest values of TAC (*p* < 0.001, *p* < 0.01, respectively). On the other hand, the RB regime promoted (*p* < 0.01) TAC in Ti-10 plants (Figure 4c).

### 2.4. Heatmap Analysis 

**Figure 5:**
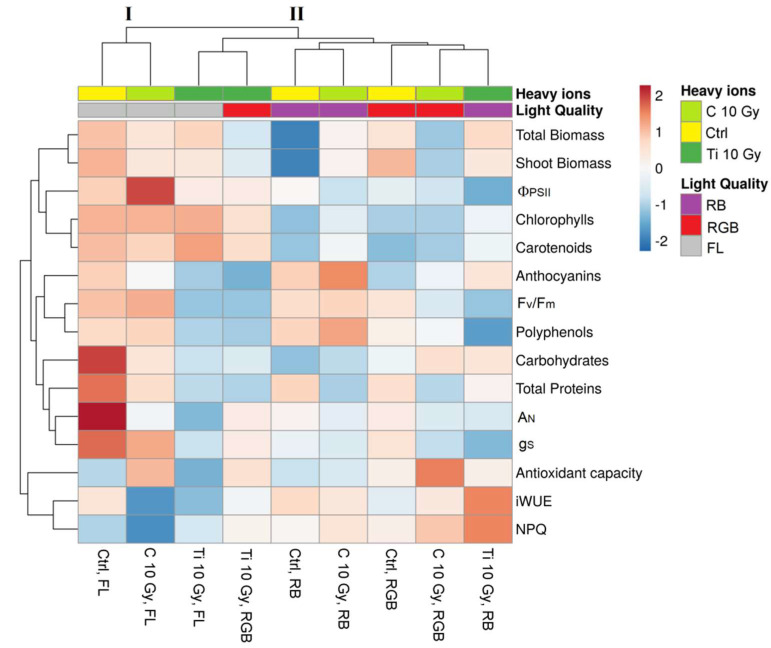
Cluster heatmap analysis summarizing morphological, eco-physiological, and biochemical parameters of *Beta vulgaris* L. cv cicla plants sprouted from Control (Ctrl) and irradiated Carbon (C-10 Gy), and Titanium (Ti-10 Gy) seeds and grown under white fluorescent (FL), red-green-blue (RGB) and red-blue (RB) LQ regimes. Numeric differences within the data matrix are shownby the color scale: light blue and dark blue indicate increasing and decreasing values, respectively. Parameters are clustered in the rows; sample groups are clustered in the columns by the two independent factors: IR treatment and LQ regimes.

An overview of all measured parameters in response to heavy ions irradiation (C-10 and Ti-10) and the three LQ regimes (FL, RGB, and RB) is reported in Figure 5.

The heatmap identified two main clusters. The first cluster (I) included plants sprouted from control and C-10-irradiated seeds grown under the FL regime. The second cluster (II) was split into two sub-clusters: the first incorporated Ti-10 irradiated plants grown under FL and RGB regimes; the second included control and C-10 plants grown under RB, control, and C-10 plants grown under RGB and Ti-10 plants grown under RB. The heatmap indicated that control and C-10 irradiated plants showed a similar response for different physiological and biochemical attributes regardless of the LQ regime. In particular, under FL, plants exhibited the highest values of biomass, pigments, PSII photochemical efficiency, and antioxidant compounds. Within the Ti-10 group, RB plants were characterized by higher values of iWUE and NPQ than FL and RGB plants. Finally, the heatmap indicated that LQ regimes exerted different physiological responses in *B. vulgaris* plants irradiated with C- or Ti-ions.

## 3. Discussion

This work highlighted that a low dose of carbon and titanium heavy ions, delivered at the seed stage, may modify the *B. vulgaris* eco-physiological response (i.e., photosynthesis and accumulation of bioactive compounds) depending on different light quality regimes during growth. These results may have implications on controlled environment agriculture, especially in extreme environments such as Space.

In the view of space cultivation, seed germination could represent a critical step. Previous research demonstrated that seed responses to IR depend on the plant species and the type of ion. The seed irradiation with C-ions at the dose of 10 Gy, reduced the germination rate in rice and bean [6,47], whereas no variation occurred in spinach for doses up to 15 Gy [48]. Conversely, there is still little information about the effect of Ti-ions on different species. Ti-ions did not affect the germination of bean seeds at the dose of 10 Gy [6]. In *B. vulgaris* seeds, the irradiation with C-ions promoted the percentage germination (100%) compared to control and Ti-ions, regardless of the LQ regimes. These results indicated that more energetic C-ions likely favored seed tegument porosity which, in turn, may have promoted germination through a higher water permeability [4,49]. In control plants, the reduction of germination under RGB and RB light regimes could be ascribed to the higher incidence of blue wavelengths, which generally inactivate the phytochrome A involved in seed germination [50]. Moreover, the higher percentage germination found in Ctrl-RGB compared to Ctrl-RB seeds may be ascribed to the presence of green wavelength, which is known to promote germination through phytochrome [51].

Generally, the exposure of plants to IR determines a reduction of plant growth and biomass, inducing a more compact plant architecture [4,52,53,54,55]. On the other hand, the RB growth regime may produce an enhancement in biomass depending on the intrinsic characteristics of the species [31,56]. In our case, IR and LQ as single factors did not affect plant biomass, but their interaction produced significant differences under RB light regime. In particular, C and Ti-RB plants were characterized by higher edible biomass than control, representing a suitable trait for plants destined to grow in BLSSs.

Heavy ions and LQ regimes and their interaction deeply affected the photosynthetic activity in *B. vulgaris*. IR generally impaired A_N_, g_S_, and iWUE, regardless of the type of radiation and dose [52,57,58,59,60]. Moreover, these parameters are strictly interconnected, as CO_2_ uptake and water use follow the same route through stomata [61]. In control plants, leaf gas exchanges were sensitive to LQ, especially under RGB and RB regimes that strongly reduced A_N_ and g_S_ compared to FL. The seed irradiation with C-ion seemed to offset the effect LQ on A_N_, which resulted comparable under all regimes. It is hypothesized that in C-ion irradiated plants, the high intensity of red and blue wavelengths of RGB and RB regimes may have improved the stomatal control, ultimately resulting in the enhancement of iWUE. However, the occurrence of stomatal limitations due to the potentially detrimental effects of C heavy ions on photosynthetic machinery cannot be excluded. On the other hand, the seed irradiation with Ti-ion under RGB and RB regimes stimulated A_N_ and g_S_ compared to FL, determining, also in this case, an improvement of iWUE. In response to different LQ regimes during growth, leaves of irradiated plants have probably adopted adjustments in mesophyll traits and stomatal movements to improve photosynthesis and iWUE [45]. According to other authors, RB wavelengths, alone or supplemented with the green light that penetrates deeper inside the canopy, may have induced changes in leaf thickness, promoting the CO_2_ diffusion within chloroplasts [56,62,63,64,65,66]. In addition, the blue wavelengths, acting on the guard cells, stimulated the stomatal opening, improving leaf conductance and consequently, photosynthesis [26,67,68,69]. 

Both IR and LQ affected not only the dark but also the light phase of photosynthesis and, more specifically, the partitioning of light energy. In RGB and RB plants, the reduction of ϕ_PSII_ was consistent with the decline of A_N_ and the rise of NPQ, indicating that the photosynthetic apparatus diverted the light energy in thermal dissipation mechanisms in conditions of reduced carbon assimilation [70]. On the contrary, in C-10-FL plants, the A_N_ decline was accompanied by high values of ϕ_PSII_. In this case, photochemical processes different from photosynthesis (i.e., photorespiration, Mehler reaction) occurred to avoid photoinhibition and photooxidative damage to photosystems. This response suggested that under C-irradiation, plants adopted a mechanism to optimize the PSII efficiency by transferring the excess light energy, which is potentially detrimental for photosystems to the other photochemical processes [71]. Control and Ti-ion-irradiated plants showed similar photochemical behavior. However, the higher NPQ measured in Ti-10 RB plants suggested that thermal dissipation processes were amplified under Ti irradiation and used as a safety valve against putative photoinhibition damages [72,73]. The absence of difference in the maximum quantum efficiency of PSII (F_v_/F_m_) among all treatments confirmed the efficiency of the different regulatory mechanisms of absorbed light induced by different heavy ion treatments.

The photochemical reactions observed in RGB- and RB-irradiated plants were consistent with the lower photosynthetic pigment content. The down-regulation of chlorophyll and carotenoid biosynthesis represents a safety strategy to avoid excessive light harvesting. Since the pigment reduction occurred in both control and irradiated plants, it may be argued that it depended on LQ more than IR. Indeed, red wavelengths, being photosynthetically more efficient, usually determined a photosynthetic pigment reduction in different species [31,66,73,74,75,76].

Our study pointed out that the FL-irradiated plants exhibited a reduced carbohydrate and protein production compared to the control consistent with the decline of photosynthesis. Previous studies performed on different plant species exposed to gamma rays demonstrated that depending on dose and plant phenological stage, the carbohydrate and protein levels may decrease, remain unchanged, or increase [13,52,77,78,79]. Generally, the higher dosage of gamma irradiation breaks down the seed proteins releasing more amino acids. This process may inhibit protein synthesis, thus inducing a decline in plant total protein content [78,80]. Besides IR, LQ can also modulate sugar and protein production. For instance, RB may induce a reduction in sugars [81] or an enhancement of the sugar and protein content in several species [31,82,83]. The intrinsic characteristics of the specific heavy ions may have produced a different behavior under the RB regime, which exerted a positive effect only when applied to Ti-ion-irradiated plants.

IR strongly influenced TPC, ANTH, and TAC, depending on ion type. While C-ions did not affect the concentration of anthocyanins and polyphenols compared to controls, Ti-ions reduced these compounds. To counteract the IR-induced oxidative stress and mitigate the risk of disease, a diet rich in polyphenols is essential for astronauts. Usually, phenolic compounds exert a screening function against high levels of solar radiation; protecting cell structures from photoinhibition damages [37,54,84,85] the same way, they offset the detrimental effects of IR [54,75,85,86,87]. The effects of IR on polyphenols and anthocyanins are controversial because some irradiated plants showed an enhancement after the exposure to gamma and X-rays or C-heavy ions [54,59,75,86], while other species exhibited a decline [54,85]. The different outcomes depend on the radiation quality and dose. In our study, Ti-ions induced a decline in polyphenols content regardless of the growth LQ regime. 

The anthocyanin concentration was not only affected by IR but also by LQ. 

In both C- and Ti-irradiated plants, the RB regime stimulated the anthocyanin synthesis compared to FL and RGB. The biosynthesis of anthocyanins is typically associated with blue light, but it may also be stimulated by red and green wavelengths [37,88]. It may be supposed that the higher intensity of red wavelengths in RB compared to the other regimes may have boosted the anthocyanin production, improving the nutritional properties of chard as observed for many crops [89,90,91]. Hence, the increase in anthocyanins may be considered a desirable feature for irradiated *B. vulgaris* plants.

Finally, C-ions irradiation determined a consistent rise of the total antioxidant capacity, which may be ascribed to the production of several different compounds characterized by antioxidant properties, as for other species, such as lettuce, irradiated with UV and gamma rays [75,92,93]. The antioxidant response of Ti-ion-irradiated plants was enhanced under RGB and RB regimes, confirming that red and blue supplemented with green wavelengths can promote the synthesis of free radical scavenger molecules. These compounds potentially improve the antioxidant capacity of chard plants and enhance their tolerance to stress conditions and nutrient quality [39,91,94,95]. Moreover, the high antioxidant content may be considered an added value, also for the use of chard as a supplement for astronauts’ diet.

## 4. Materials and Methods

### 4.1. Plant Material, Irradiation Procedure, and Experimental Design

*Beta vulgaris* L. var. *cicla* (white chard) is a widely cultivated crop, considered a functional food because of its high content of secondary metabolites, associated with some beneficial effects, including anti-tumoral activity [96]. Moreover, its compactness and high ratio of edible biomass/wastes make chard one of the candidates’ crops to be cultivated in the Space Greenhouses, designed as Closed Ecological Life Support Systems [3].

Dry chard seeds (*n* = 150) were divided into not-irradiated control (*n* = 50) and treated groups (*n* = 100). More specifically, 50 seeds were irradiated with carbonium (isotope ^12^C; E: 300 MeV/u (monoenergetic), LET: 13 keV/µm; dose rate 1 Gy/min), and 50 seeds with titanium (isotope ^50^Ti; E: 1000 MeV/u (monoenergetic), LET: 108 keV/µm; dose rate 1 Gy/min) at the dose of 10 Gy. In particular, C-ions are considered reference radiation and the dose of 10 Gy, being below the threshold for DNA damage, may not be lethal for plant development [19,97]. Seeds were collected into T25-flasks and the irradiation was performed using a pencil beam in a spread-out Bragg peak (SOBP), at the heavy-ion synchrotron (SIS) at the GSI Helmholtz zentrumfür Schwerionenforschung GmbH, (Darmstadt, Germany).

**Figure 6:**
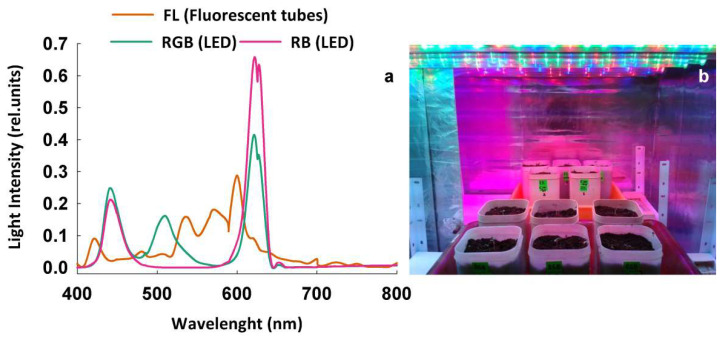
(**a**) Spectral distributions in the relative energy of the light quality regimes used in the study: FL—white fluorescent tubes; LED RGB—red–green–blue; LED RB—red–blue. Photon flux density: 300 µmol m^−2^s^−1^; (**b**) Particular of growth chambers with RGB (front) and RB (back) light quality regimes.

The seeds were maintained in the same storage and transport facilities to avoid bias due to different conditions during traveling. Irradiated and not-irradiated (control) seeds were then transferred to the laboratory and placed in Petri dishes on three layers of filter paper to follow the germination process. Both germination and plant cultivation took place in a climatized room under three different light regimens: (1) FL, provided by white fluorescent tubes (Lumilux L360W/640 and L360W/830, Osram, Germany); (2) RGB (red–green–blue) and (3) RB (red–blue) provided by red, green, and blue LEDs (Octa Light LTD, Bulgaria) with the following emission peaks: 630 nm red, 510 nm green, 440 nm blue. The spectral composition of LQ treatments (Figure 6a,b) was measured by a SR-3000A spectroradiometer at 10 nm resolution (Macam Photometrics Ltd., Livingston, Scotland, UK) at the top of the canopy.

The total photosynthetic photon flux density (PPFD) was fixed at 300 ± 5 µmol photons m^−2^s^−1^ at the canopy level for all LQ regimes. All plants were kept under air temperature of 25/20 °C (day/night), relative humidity 60–70%, and a photoperiod of 12 h. Plants were fertilized with tap water and Hoagland’s solution every two weeks.

The plant growth was followed up to 60 days after sowing (DAS) at the Plant Physiology and Genetics Institute of the Bulgarian Academy of Science (Sofia, Bulgaria). Gas exchanges and fluorescence emission measurements were carried out 60 days after sowing (DAS) on fully expanded leaves to assess how radiation may have affected the functionality of the photosynthetic apparatus and if the plant growth under different LQ may have influenced the plant photosynthetic behavior. In addition, at the end of the vegetative cycle (60 DAS), biometrical measurements, leaf functional traits, photosynthetic pigments, total carbohydrates, and antioxidant content were also determined on mature leaves as a proxy for carbon allocation and plant nutritional status. These analyses were performed at the Department of Biology of University Federico II of Naples (Italy). 

**Figure 7:**
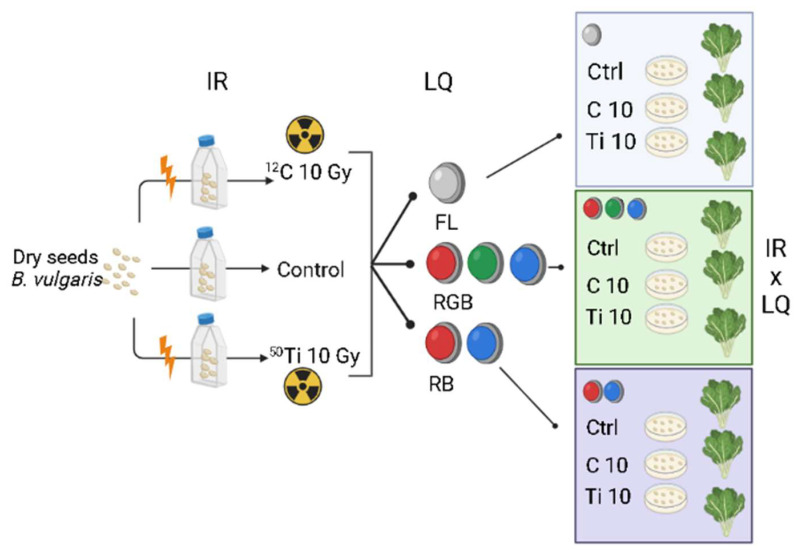
Schema of the experimental design created with BioRender.com (accessed on 2 May 2022).

Figure 7 displays the schema of the experimental design of the study.

### 4.2. Germination and Biometrical Measurements

The percentage of seed germination under FL, RGB, and RBLQ regimes was evaluated. Seeds were considered germinated when the root protruded from the seed coat. The percentage germination (GP) was calculated at 7 days after sowing (DAS), according to the formula:

GP_7DAS_ = [(Number of germinated seeds after 7 days)/Total seed number] × 100 (1)

After germination, 10 control, 10 C—ion, and 10 Ti—ion—irradiated seedlings were sown in 3.0 L pots filled with peat soil. At the end of the experimental period, 60 DAS, total biomass (TB), and shoot biomass (SB) were determined on five plants for each treatment, weighing the whole plant and the shoot portion, respectively. The TB and SB were expressed as grams of fresh weight per plant (g FW plant^−1^).

### 4.3. Leaf Gas Exchange and Chlorophyll aFluorescence Emission Measurements

Leaf gas exchange was measured at 60 DAS on five fully expanded leaves from five plants per treatment (one leaf per plant) by a portable gas-exchange system (LCpro+, ADC BioScientific, UK). The middle part of the leaf was clamped into the 6.25 cm^2^ gas-exchange cuvette and exposed to a constant flow of 300 μmol s^−1^ of synthetic air (79% N_2_, 21% O_2_, and 400 µmol mol^−1^ CO_2_). Measurements were carried out at 25 ± 2 °C leaf temperature and 500 µmol m^−2^s^−1^ photosynthetic photon flux density (PPFD). The relative humidity in the leaf chamber was set at 50–60%. The intrinsic water use efficiency (iWUE) was calculated as a ratio between photosynthesis (A_N_) and stomatal conductance to water (g_s_). All gas-exchange parameters were recorded after reaching a steady-state, usually 7–10 min for each measurement, and calculated by the equations of von Caemmerer and Farquhar [98] with the software operating within the gas-exchange system.

Chlorophyll *a* fluorescence measurements were carried out on five fully expanded leaves from five plants per treatment (one leaf per plant) using a Fluorescence Monitoring System (FMS, Hansathech Instruments, King’s Lynn, UK). The determination of minimum (F_o_) and maximum (F_m_) fluorescence was carried out on 30 min dark adapted leaves. The maximum quantum yield of PSII photochemistry (F_v_/F_m_) was determined as (F_m_−F_o_)/F_m_. The measurements in the light were carried out on leaves adapted to PPFD of 500 µmol m^−2^ s^−1^. A saturating pulse of 0.8 s with >6000 µmol photons m^−2^ s^−1^ was applied to determine the maximum (F_m_’) and the steady-state (F_s_) fluorescence in light adapted conditions. The quantum yield of PSII electron transport (ϕ_PSII_) was calculated according to Genty et al. [99] as: ϕ_PSII_ = (F_m_’ − F_s_)/F_m_’. The non-photochemical quenching (NPQ) was calculated as NPQ = (F_m_− F_m_’)/F_m_’ [100].

### 4.4. Determinationof Photosynthetic Pigments and Antioxidants 

Photosynthetic pigment and antioxidant contents were determined on five fresh leaves collected from five different plants (one leaf per plant) from each experimental condition. 

The determination of the photosynthetic pigments content, namely total chlorophylls (*a* + *b*) and carotenoids (*x* + *c*), was performed according to Lichtenthaler [101]. Leaf samples of the known area (0.283 cm^2^) were homogenized in ice-cold 100% acetone using a mortar and pestle. The extracts were centrifuged at 5000 rpm for 5 min in a Labofuge GL (Heraeus Sepatech, Hanau, Germany). The sample absorbance was measured by a spectrophotometer (UV-VIS Cary 100; Agilent Technologies) at wavelengths of 470, 645, and 662 nm. Pigment concentration was expressed as µg cm^−2^. 

The total polyphenol content was evaluated on fresh samples powdered in liquid nitrogen. Samples were extracted in 80% methanol at 4 °C, centrifuged at 11,000 rpm for 5 min. The soluble fraction was mixed with 10% Folin–Ciocâlteu solution, 1:1 *v*/*v*, and after 3 min, 700 mM Na_2_CO_3_ solution was added to the resulting mixture (1:5, *v*/*v*). Samples were incubated for 2 h in the darkness. The absorbance was measured at 765 nm with a spectrophotometer (UV-VIS Cary 100; Agilent Technologies). The total polyphenol amount was expressed as mg of Gallic Acid Equivalents g^−1^ FW (mg GAE g^−1^ FW) using a gallic acid standard curve.

The anthocyanin content was determined on fresh samples powdered in liquid nitrogen, treated with methanol 1% HCl solution and stored overnight at 4 °C. After adding 1:0.6 (*v*/*v*) ultra-pure water and chloroform at 1:1.6, *v*/*v*), samples were centrifuged at 11,000 rpm for 5 min. After mixing the supernatant with 1:1 (*v*/*v*) (60% (methanol 1% HCl) 40% ultra-pure water)solution, the absorbance was measured using a spectrophotometer (UV-VIS Cary 100, Agilent Technologies, Palo Alto, CA, USA) at 530 and 657 nm. The relative amount of anthocyanin was expressed as (A_530_—1/3A_657_) g^−1^ FW [102]. 

The antioxidant capacity was assessed by the Ferric Reducing Antioxidant Power (FRAP) assay, performed on fresh leaves powdered in liquid nitrogen, according to George et al. [103]. Briefly, the samples (0.250 g) were treated with methanol/water solution (60:40, *v*/*v*) and centrifuged at 14,000 rpm for 15 min at 4 °C. The extracts were mixed with the FRAP reagents (300 mM acetate buffer pH 3.6, 1:16.6 *v*/*v*; 10 mM tripyridyltriazine, TPTZ, in 40 mM HCl, 1:1.6 *v*/*v*; 12 mM FeCl_3_, 1:1.6 *v*/*v*) and incubated for 1 h in the dark. Then, the absorbance was read at 593 nm by a spectrophotometer (UV-VIS Cary 100; Agilent Technologies). The antioxidant capacity was calculated using a Trolox standard curve and expressed as µmol Trolox equivalents g^−1^ FW (µmol Trolox eq g^−1^ FW).

### 4.5. Total Soluble Carbohydrate Content and Protein Quantification

Total soluble carbohydrates were determined on five leaves for each treatment by the anthrone method, as reported in Hedge and Hofreiter [104]. The absorbance was measured at 630 nm using a spectrophotometer (UV-VIS Cary 100, Agilent Technologies, Palo Alto, CA, USA). The amount of soluble carbohydrates in the extracts was expressed as mg Glucose equivalents g^−1^ FW (mg Glu eq g^−1^ FW) using a Glucose standard curve.

Protein extraction was carried out on five fresh leaf samples ground in liquid nitrogen, according to Wang et al. [105]. Total protein content was quantified by Bradford colorimetric assay [106], measuring the sample absorbance at 595 nm by a spectrophotometer (UV-VIS Cary 100, Agilent Technologies, Palo Alto, CA, USA). Using a BSA standard curve, the protein concentration was expressed as µg BSA (bovine serum albumin) equivalents g^−1^ FW.

### 4.6. Statistical Analysis

Statistical data analysis was performed using SigmaPlot 12 software (Jandel Scientific, San Rafael, CA, USA). The effect of IR (C- and Ti-ions) and LQ regimes (FL, RGB, RB) on morphological, ecophysiological, and biochemical traits of chard plants were evaluated by processing data by two-way analysis of variance (ANOVA) followed by Duncan multiple comparison tests (*p* < 0.05). The Kolmogorov–Smirnov test was used to check the normality. When the interaction between the two factors (IR×LQ) was significant, data were further processed by applying one-way ANOVA, and multiple comparison tests were done with the Duncan test. 

The overall parameters were visualized by a heatmap, plotted using the ClustVis program package (https://biit.cs.ut.ee/clustvis/online, accessed on 20 June 2022) and clustering both rows and columns with Euclidean distance and average linkage. In the heatmap, the numeric differences were evidenced by a color scale: red and blue indicate increasing and decreasing values, respectively. 

## 5. Conclusions

The irradiation of dry chard seeds with carbon or titanium high-energy ions significantly modified the plant response to light quality. In particular, under the FL regime, gas exchanges of C- and Ti-ion-irradiated plants strongly declined compared to control. However, control and C-ion-irradiated plants showed a physiological performance higher than titanium plants in terms of for pigments content, PSII photochemical efficiency, and bioactive compounds.

The growth under RGB and RB regimes offset the differences of gas exchanges between control and C- and Ti-ion plants. C-ions induced the strongest antioxidant response regardless of light quality regimes. Furthermore, the interaction Ti-ion × RB was effective in improving iWUE, and the production of pigments, carbohydrates, and antioxidants. 

The overall results indicate that by manipulating the interaction IR × LQ, it is possible to regulate the photosynthesis in order to obtain plants that are more performing in resource regeneration linked to gas exchanges (CO_2_ removal, O_2_ production) but also to modify the bioactive compound amounts in leaf edible tissues, which may result in a beneficial outcome for the astronauts’ diet.

## Figures and Tables

**Table 1:** Analysis of variance and comparison of means for total biomass (TB) and shoot biomass (SB) of *B. vulgaris* plants sprouted from Control (Ctrl) and irradiated Carbon (C-10 Gy) and Titanium (Ti-10 Gy) seeds, and grown under white fluorescent (FL), red–green–blue (RGB) and red–blue (RB) light quality regimes.

	TB	SB
**IR**		
Ctrl	25 ^a^	21 ^a^
C-10	27 ^a^	21 ^a^
Ti-10	27 ^a^	21 ^a^
**LQ**		
FL	29 ^a^	23 ^a^
RGB	24 ^a^	19 ^a^
RB	26 ^a^	21 ^a^
**IR × LQ**		
Ctrl-FL	31 ^a^	25 ^a^
Ctrl-RGB	28 ^a^	24 ^a^
Ctrl-RB	17 ^b^	14 ^b^
C 10-FL	28 ^a^	22 ^a^
C 10-RGB	25 ^a^	21 ^a^
C 10-RB	26 ^a^	21 ^a^
Ti 10-FL	30 ^a^	22 ^a^
Ti 10-RGB	23 ^a^	19 ^a^
Ti 10-RB	29 ^a^	22 ^a^
**Significance**		
IR	NS	NS
LQ	NS	NS
IR × LQ	*	*

TB—Total biomass, g FW plant^−1^; SB—Shoot biomass, g FW plant^−1^. Different letters in each column indicate significant differences according to Duncan’s test (*p* < 0.05). NS—not significant; * *p* < 0.05.

**Table 2:** Analysis of variance and comparison of means for net CO_2_ assimilation (A_N_), stomatal conductance to water (g_S_), intrinsic water use efficiency (iWUE), quantum yield of PSII electron transport, ϕ_PSII_, non-photochemical quenching (NPQ), maximum PSII photochemical efficiency, (F_v_/F_m_) of *B. vulgaris* plants sprouted from Control (Ctrl) and irradiated Carbon (C-10 Gy) and Titanium (Ti-10 Gy) seeds, and grown under white fluorescent (FL), red–green–blue (RGB) and red–blue (RB) light quality regimes.

	A_N_	g_s_	iWUE	ϕ_PSII_	NPQ	F_v_/F_m_
**IR**						
Ctrl	9.1 ^a^	0.16 ^a^	59 ^a^	0.34 ^a^	2.4 ^b^	0.76 ^a^
C-10	6.7 ^b^	0.13 ^b^	53 ^a^	0.34 ^a^	2.7 ^a^	0.75 ^a^
Ti-10	6.4 ^b^	0.13 ^b^	56 ^a^	0.29 ^a^	2.8 ^a^	0.74 ^a^
**LQ**						
FL	7.9 ^a^	0.16 ^a^	48 ^b^	0.38 ^a^	2.2 ^b^	0.75 ^a^
RGB	7.4 ^b^	0.14 ^b^	55 ^b^	0.31 ^b^	2.8 ^a^	0.75 ^a^
RB	6.8 ^c^	0.12 ^c^	65 ^a^	0.28 ^b^	2.9 ^a^	0.75 ^a^
**Significance**						
IR	***	***	NS	NS	**	NS
LQ	*	***	**	*	***	NS
IR × LQ	***	***	NS	NS	***	NS

A_N_—net CO_2_ assimilation (µmol CO_2_ m^−2^ s^−1^); g_S_—stomatal conductance to water (mol H_2_O m^−2^s^−1^), iWUE—intrinsic water use efficiency (µmol CO_2_ mol^−1^ H_2_O); ϕ_PSII_—quantum yield of PSII electron transport; NPQ—non-photochemical quenching; F_v_/F_m_—maximum PSII photochemical efficiency. Different letters in the columns indicate significant differences according to Duncan’s test (*p* < 0.05). NS—not significant; * *p* < 0.05; ** *p* < 0.01; *** *p* < 0.001.

**Table 3:** Analysis of variance and comparison of means for chlorophylls (CHL), carotenoids (CAR), carbohydrates (CARB), proteins (PROT), polyphenols (POL), anthocyanins (ANTH), and total antioxidant capacity (TAC) of *B. vulgaris* plants sprouted from Control (Ctrl) and irradiated Carbon (C-10 Gy) and Titanium (Ti-10 Gy) seeds, and grown under white fluorescent (FL), red–green–blue (RGB) and red–blue (RB) light quality regimes (*n* = 5).

	CHL	CAR	CARB	PROT	POL	ANTH	TAC
**IR**							
Ctrl	38 ^b^	5.7 ^b^	36 ^a^	480 ^a^	0.91 ^a^	2.8 ^a^	2.8 ^c^
C-10	41 ^b^	6.7 ^b^	33 ^b^	347 ^b^	0.94 ^a^	2.9 ^a^	4.1 ^a^
Ti-10	47 ^a^	8.4 ^a^	31 ^b^	321 ^b^	0.47 ^b^	2.0 ^b^	3.3 ^b^
**LQ**							
FL	54 ^a^	8.8 ^a^	42 ^a^	427 ^a^	0.81 ^a^	2.5 ^b^	3.0 ^b^
RGB	37 ^b^	5.7 ^b^	33 ^b^	337 ^b^	0.70 ^b^	1.9 ^c^	4.3 ^a^
RB	36 ^b^	5.9 ^b^	26 c	381 ^a^	0.80 ^a^	3.3 ^a^	2.9 ^b^
**Significance**							
IR	***	***	*	***	***	***	***
LQ	***	***	***	*	*	***	***
IR × LQ	***	***	***	**	**	NS	***

CHL—Chlorophylls (µg cm^−2^); CAR—Carotenoids (µg cm^−2^); CARB—Carbohydrates (mg GLU eq g^−1^ FW); PROT—Proteins (µg BSA eq g^−1^ FW); POL—Polyphenols (mg GAE g^−1^ FW); ANTH—Anthocyanins ((A_530_-1/3A_657_) g^−1^ FW); TAC—Total antioxidant capacity (µmol Trolox eq g^−1^ FW). Different letters in each column indicate significant differences according to Duncan’s test (*p* < 0.05). NS—not significant; * *p* < 0.05; ** *p* < 0.01; *** *p* < 0.001.

## Data Availability

The data supporting the results of this study are accessible from the corresponding author (C.A. (Carmen Arena)) upon reasonable request.
